# The effects of cloth face masks on cardiorespiratory responses and VO_2_ during maximal incremental running protocol among apparently healthy men

**DOI:** 10.1038/s41598-022-26857-w

**Published:** 2022-12-24

**Authors:** Takeshi Ogawa, Jun Koike, Yuka Hirano

**Affiliations:** 1 Division of Art, Music and Physical Education, Osaka Kyoiku University, Osaka, 582-8582 Japan; 2 Department of Education, Osaka Kyoiku University, Osaka, 582-8582 Japan

**Keywords:** Physiology, Circulation, Respiration

## Abstract

We aimed to determine the effects of wearing a cloth face mask on cardiorespiratory response, peak oxygen uptake (*V*o_2_), respiratory muscle effort, and exercise tolerance during incremental exercise. The study had a randomized crossover design: 11 apparently healthy young men performed the Bruce protocol treadmill test in two conditions, wearing a cloth face mask (CFM) and without CFM (CON), in random order. Minute ventilation and oxygen uptake were measured using a mass spectrometry metabolic analyzer; cardiac output (CO) was measured using an impedance CO monitor; and mouth pressure (P_m_) was measured and calculated as an integral P_m_ to assess respiratory muscle effort. Maximal minute ventilation was 13.4 ± 10.7% lower in the CFM condition than in the CON condition (*P* < 0.001). The peak *V*o_2_ (52.4 ± 5.6 and 55.0 ± 5.1 mL/kg/min in CFM and CON, respectively) and CO were not significantly different between the two conditions. However, the integral value of P_m_ was significantly higher (*P* = 0.02), and the running time to exhaustion was 2.6 ± 3.2% lower (P = 0.02) in the CFM condition than in the CON condition. Our results suggest that wearing a cloth face mask increased respiratory muscle effort and decreased ventilatory volume in healthy young men; however, *V*o_2_ remained unchanged. Exercise tolerance also decreased slightly.

## Introduction

The novel coronavirus disease (COVID-19) spreads mainly through exposure to droplets while breathing, coughing, and sneezing; therefore, the World Health Organization (WHO) has recommended wearing a face mask covering the nose and mouth to prevent COVID-19 infection transmission^1,2^. The spread of droplets is greater during exercise because of the vigorous breathing involved^3,4^. Thus, WHO recommended social distancing (> 1 m) while resting and exercising. However, wearing a face mask is not recommended during vigorous physical activity^1^.

Several studies have examined the impact of N95 respirators and surgical masks^5–12^. It has been noted that wearing a surgical mask or N95 respirator during exercise may increase discomfort and decrease exercise tolerance^7–9^. Conversely, some studies reported that wearing a surgical mask had no effect on dyspnea, pulmonary gas exchange, or exercise performance^10–12^. The primary effects of wearing a face mask on the physiological responses during exercise include increased respiratory resistance and dead space, resulting in impaired gas exchange due to hypoxia and carbon dioxide rebreathing^13,14^. Increased airflow resistance when wearing a face mask results in decreased pulmonary ventilation^5,6^. Inadequate hyperventilation during intensive exercise can lead to decreased arterial oxy-hemoglobin saturation (SaO_2_)^15^. Two studies have reported a decrease in the maximal oxygen uptake (*V*o_2max_) with the use of surgical masks, thereby decreasing exercise tolerance^6,8^.

Furthermore, the highest minute ventilation (*V*_E_) during high-intensity exercise increases the work of breathing (Wb), resulting in a preference for blood flow to the respiratory muscles, which can consequently compromise the blood flow to active muscles^16,17^ and subsequently limit exercise tolerance^18^. It is believed that as the filter flow resistance slightly increases with a constant airflow of a face mask, the resistive Wb would not increase even during high-intensity exercise^14^. However, humans do not breathe at a constant flow rate; hence, Wb during intensive exercise might be greater while wearing a face mask. Therefore, whether wearing a face mask during exercise has physiological disadvantages or health risks is intriguing and debatable.

N95 respirators are commonly used by medical professionals in the workplace and are unlikely to be used in sports activities. Surgical masks are occasionally used during sports activities^1^; however, recently, cloth face masks designed for use during exercise have become available. A cloth face mask is expected to have a lower airflow resistance than a surgical mask or N95^14^. Therefore, we hypothesized that wearing a cloth face mask would have no substantial effect on cardiorespiratory response and respiratory muscle activity during exercise; however, its effect on oxygen uptake (*V*o_2_) and thereby, exercise tolerance, remains unclear. Thus, the primary objective of this study was to examine the effect of wearing a cloth face mask while exercising on the cardiorespiratory response during incremental running, and the secondary objective was to examine the mouth pressure and *V*o_2_ during the exercise.

## Methods

### Ethical issue

This study was conducted in accordance with the Declaration of Helsinki, and the experiments were performed taking ethics, human rights, and the protection of personal information into consideration. This study was approved by the ethics committee of Osaka Kyoiku University (Approval number: 21051). All the participants signed a written informed consent before participating in this study.

### Participants

The study participants included university students of physical education on campus. Thus, they were physically active. The inclusion criteria were as follows: participants were aged 18 years or older, fully understood the experiment, and gave their written consent to participate. We recruited participants by canvassing within the university, and as a result, they were young. The exclusion criteria were heart disease history, current arrhythmia, chest pain, exercise pain, and respiratory disease history. Forty individuals participated in a briefing session. Before the study commenced, the purpose and potential risks were carefully explained. Subsequently, 16 participants who volunteered to participate answered questions regarding their respiratory and cardiovascular disease history using the Physical Activity Readiness Questionnaire^19^. All the participants were nonsmokers and had no history of medical illnesses. The sample size was calculated using G*power 3.1, based on a previous study, assuming that *V*O_2peak_ corresponded to 32.2 ± 9.0 and 43.9 ± 8.1 mL/kg/min with and without a cloth face mask^20^, with a 5% significance level and 90% power. Therefore, it was estimated that eight participants would be required.

### Study design

**Figure 1:**
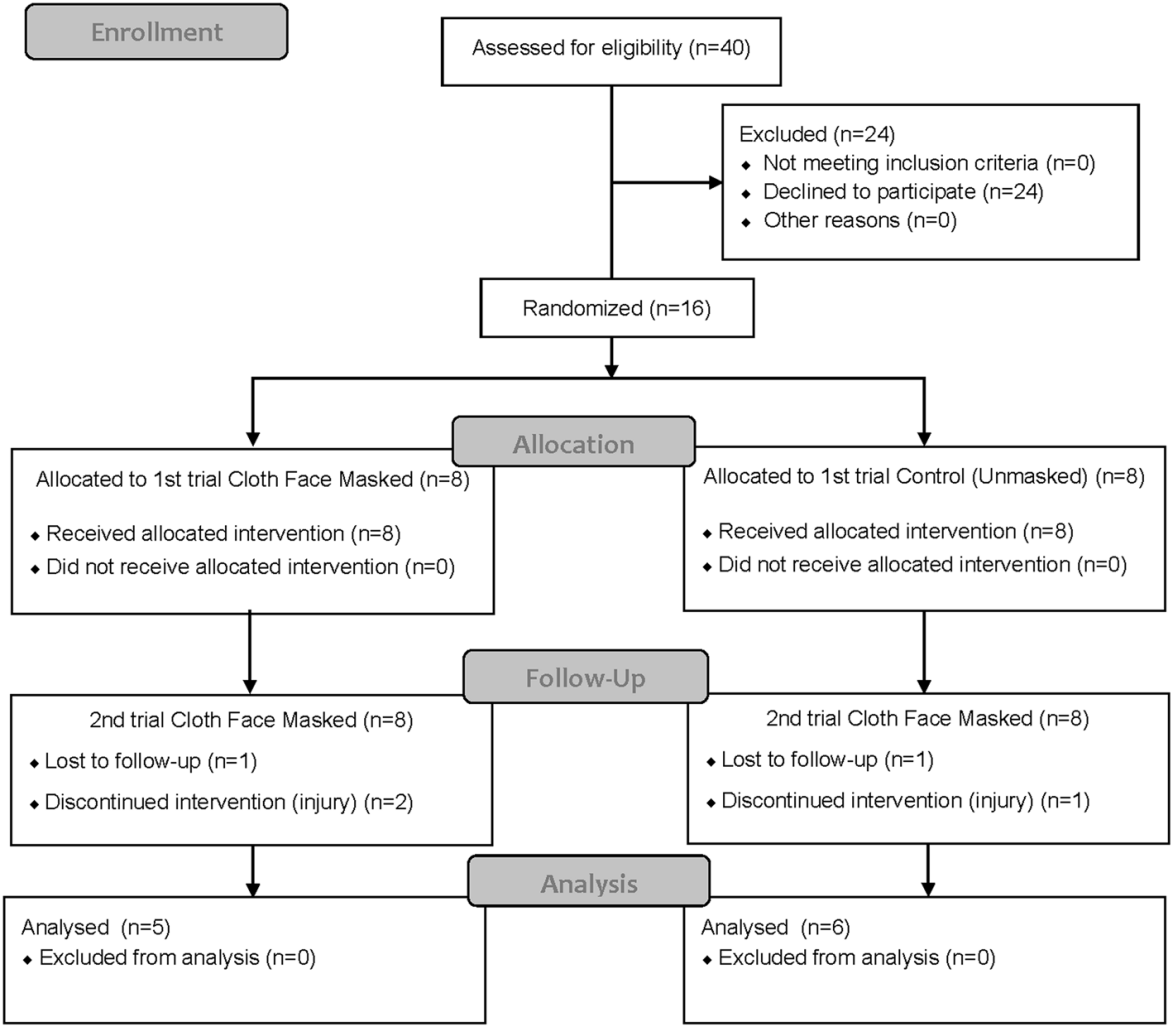
CONSORT flow diagram. The diagram indicates how many individuals were screened and completed under two condition trials.

Toward this goal, 11 healthy young men underwent an incremental load treadmill running test until exhaustion with and without cloth face masks. In this study, a randomized crossover design was employed. All the participants underwent an incremental treadmill load running test until exhaustion under two conditions: with a cloth face mask (CFM) and without a mask (CON) in random order. Each test was conducted on a separate day, in a random order, and at least 48 h apart. To minimize daily variations, both test conditions were conducted at the same time of the day for each participant within a 2-h time difference. Participants were briefed on the experimental procedures and practiced the test protocol 1 week before the study to familiarize themselves with the equipment and exercise protocol. After familiarization, participants were randomized into two groups and underwent the first running test. The second test was performed under different conditions from the first trial (Fig. 1). Participants were instructed not to consume caffeine or alcohol and not to engage in heavy exercise for 24 h prior to the test. The participants had their height and weight measured and performed voluntary stretching exercises on the test day. A 3-min warm-up was then performed by walking on a treadmill (3.0 km/h with 0% incline). After the warm-up, the participants attached a one-way expiratory mask (601M, ARCO, Chiba, Japan) connected to a mass spectrometer sensor via a pipe for expiratory gas analysis. Six ECG electrodes (Vitorode M-150, Nihon Kohden, Tokyo, Japan) were attached to measure the cardiac output (CO). To prevent falling, the participants were fitted with an upper-body harness. The test was then initiated, and the participants had to rest for 3 min before starting the exercise to measure the resting values. The experiment was conducted in October. The room temperature was controlled using an air conditioner; nonetheless, the room windows had to be opened in accordance with the university's COVID-19 prevention guidelines. The room temperature was 25.0 ± 0.5 °C for all the tests.

### Face mask

In the CFM condition, a cloth face mask (DESCENTE Athletic Mask, DESCENTE, Osaka, Japan) was used (outer lining: 100% polyester, inner lining: 98% polyester, 2% polyurethane). Rizki and Kurniawan^21^ reported that cloth face masks can filter the air to a certain extent, and the polyester cloth face masks provides the most efficient filtration. Hence, the cloth face mask used in this study was expected to prevent droplet dispersal to some extent. After the face mask was attached, an expiratory mask for gas analysis was placed over it and secured with straps to prevent gas leakage. Before starting the test, the participants engaged in expiratory efforts until a positive mouth pressure of 50 cmH_2_O was detected to verify any gas leakage. Positive pressure was generated by shutting the gas pipe outlet connected to the expiratory mask (601M, ARCO, Chiba, Japan) with hands. Leakage was carefully checked for via sound, sensory, and visual inspections (such as whether the mask was lifted and whether air flowed from the side).

### Exercise protocol

The Bruce treadmill protocol^22^ was used for the graded load exercise test. We adopted the Bruce protocol because several previous studies^6,8,20,23^ employed it in their treadmill exercise tests. Treadmill speed and incline were increased every 3 min after the onset of exercise until exhaustion was reached (Table 1). The criterion for exhaustion was the point at which the participant could not maintain the running speed and dropped by > 0.8 m. The participant was provided with verbal encouragement during the exercise.

**Table 1:** Treadmill test protocol.

Stage	Velocity (km/h)	Incline (%)
1	2.7	10
2	4.0	12
3	5.4	14
4	6.7	16
5	8.0	18
6	8.8	20
7	9.6	22

### Measurements

Respiratory and metabolic responses were continuously measured during the exercise by analyzing expiratory gases using a mass spectrometer (ARCO-2000N, ARCO, Chiba, Japan) connected to an expiratory mask through a silicone pipe. Maximal oxygen uptake (*V*o_2_), carbon dioxide elimination (*V*co_2_), tidal volume (VT), respiratory frequency (*f*_R_), minute ventilation (*V*_E_), alveolar ventilation (*V*_A_), *V*_E_ /*V*o_2_, *V*_E_ /*V*co_2_, and end-tidal partial pressure of Co_2_ (P_ETCO2_) were measured. The mass spectrometer was calibrated using two gases (ambient air equivalent O_2_, 20.93%; CO_2_, 0.05%; N_2_, balance and expired gas equivalent O_2_, 13.0%; CO_2_, 5.01%; N_2_, balance). To ensure that the *V*o_2_ reached the maximum, participants met at least three of the following criteria: (1) a respiratory exchange ratio of ≥ 1.10 (43% of trials), (2) heart rate (HR) that reached 90% of the age-predicted maximal heart rate (220−age) (100% of trials), (3) rate of perceived exertion (RPE) of > 16 (100% of trials), and (4) the participant was unable to continue the exercise (100% of trials). (5) The *V*o_2_ plateaued: a *V*o_2_ plateau was the deviation from the extrapolated *V*o_2_-time linear regression using 30 s data (the actual value was < 400 mL/min from the extrapolated value)^24^ (50% of trials). All the parameters were averaged every 60 s for analysis.

The cardiac response was measured using an impedance CO monitor (PhysioFlow Q-Link, Manatec Biomedical, Paris, France). HR, stroke volume (SV), and CO were calculated for each beat and averaged every 60 s for analysis.

Mouth pressure was measured by fixing a catheter tip pressure transducer (MicroSensor Basic Kit, Codman & Shurtleff, Inc., MA, USA). The catheter was covered with a plastic tube (diameter: 4 mm, length: 250 mm) and fixed with surgical tape from the nasal dorsum to the nasal apex to prevent the mask from contacting the sensor portion at the tip of the catheter. When wearing the face mask and expiratory mask, it was confirmed that the tip did not touch the skin or mask. The catheter tip pressure transducer was calibrated by immersing the catheter in a light-shielding pipe filled with warm water (37 °C) to a depth of 0–60 cm before the experiment to obtain a calibration signal. Mouth pressure was recorded on a laptop (Dynabook EX/55, TOSHIBA, Tokyo, Japan) at a sampling frequency of 200 Hz via an AD converter (PowerLab 8a/d, AD Instruments, Sydney, Australia) and analyzed using a waveform analysis software (Lab Chart ver. 7, AD instrument, Sydney, Australia). The absolute values were integrated from the obtained mouth pressure data and used as ∫Pm.

SaO_2_ was measured using a pulse oximeter (SpO_2_) (N-560, Covidien Med, Dublin, Ireland) placed on the forehead, which was recorded every minute.

The RPE was measured using the Borg scale^25^, and dyspnea was measured using the modified Borg scale^26^ by asking the participant every minute.

### Statistical analysis

All the variables obtained in this study are presented as the mean ± standard deviation. All statistical analyses were performed using SPSS 28 for Mac (IBM, NY, USA). Normality was tested using the Shapiro–Wilk test. A paired *t*-test was used to compare the CFM and CON variables at maximal exercise intensity (*V*o_2peak_, *V*co_2peak_, VT, *f*R, *V*_E_, *V*_A_, *V*_E_/*V*o_2_, *V*_E_/*V*co_2_, P_ET_co_2_, SV, HR, CO, ∫Pm, and SpO_2_) and time to exhaustion. Cohen’s d (d) was used for the effect size in the pairwise tests, and the effect size was determined as small, medium, or large for effect sizes exceeding 0.2, 0.5, and 0.8, respectively. Repeated measurements of two-way analysis of variance (Stage × Mask) were used for the last-minute mean values of each stage for *V*o_2_, *V*co_2_, VT, *f*_R_, *V*_E_, *V*_A_, *V*_E_/*V*o_2_, *V*_E_/*V*co_2_, P_ET_co_2_, SV, HR, CO, ∫Pm, SpO_2_, RPE, and dyspnea. The Bonferroni method was used to adjust for multiple comparisons. For the effect size, the ηp^2^ was used to analyze variance, and the effect size was determined to be small, medium, and large for effect sizes of 0.01, 0.06, and values exceeding 0.14, respectively. The significance level was set at 5%.

### Ethics declarations

This study was conducted in accordance with the Declaration of Helsinki, and the experiments were performed taking ethics, human rights, and the protection of personal information into consideration. This study was approved by the ethics committee of Osaka Kyoiku University (Approval number: 21051). Participants signed a written informed consent before participating in this study.

## Results

After commencing the experimental period, three participants had injuries in their daily lives, and two could not spare time for the experiments owing to unexpected reasons. Finally, 11 participants completed the tests [mean age: 21.3 ± 2.0 years, mean height: 175.3 ± 5.9 cm, and mean weight: 68.4 ± 3.4 kg].

Two of the eleven participants who completed the experiment and failed to measure mouth pressure were excluded from the analysis of the integrated absolute value of mouth pressure (∫Pm).

Table 2 presents variables for the maximal values of the incremental treadmill running test. No significant difference was observed in the *V*O_2peak_ between the CFM and CON conditions (52.4 ± 5.8 and 55.0 ± 5.1 mL/kg/min in the CFM and CON conditions, respectively, P = 0.21); however, the extent of *V*O_2peak_ decrease was 4.4 ± 11.4% in the CFM condition. *V*_Epeak_ was 13.4 ± 10.7% lower in the CFM than in the CON condition (*P* = 0.002, d = 1.24). The tidal volume (VT) was not significantly different between the CFM and CON conditions; nonetheless, respiratory frequency (*f*_R_) was 6.9 ± 11.2% lower in the CFM condition than in the CON condition (*P* = 0.04, d = 0.61). Alveolar ventilation (*V*_A_) was also 13.4 ± 11.0% lower in the CFM condition than in the CON condition (*P* < 0.003, d = 1.19). *V*_E_/VO_2_ and *V*_E_/*V*CO_2_ were significantly lower in the CFM condition than in the CON condition (*P* < 0.001, d = 1.69; *P* < 0.001, d = 1.86, respectively). The end-tidal partial pressure of carbon dioxide (P_ET_co_2_) was significantly higher in the CFM condition than in the CON condition (*P* < 0.004, d = 1.13). However, SpO_2_ was not significantly different between the CFM and CON conditions. There were no significant differences in cardiac variables between CFM and CON conditions. ∫Pm was 20.7 ± 22.6% higher in the CFM condition than in the CON condition (*P* = 0.02, d = 0.95). Furthermore, the time to exhaustion decreased by 2.6 ± 3.2% in the CFM condition compared to the CON condition (*P* = 0.02, d = 0.40).

**Table 2:** Variables for cardiorespiratory responses for *V*O_2peak_ intensity.

	CFM	CON	Effect size (*d*)	*P* value
*V*o_2peak_ (mL/min)	3601 ± 342	3795 ± 457	0.40	0.20
*V*o_2peak_ (mL/min/kg)	52.4 ± 5.8	55.0 ± 5.1	0.42	0.21
*V*co_2peak_ (mL/min)	3810 ± 359	4128 ± 488	0.64	0.06
VT (L)	2.2 ± 0.3	2.3 ± 0.3	0.54	0.11
*f*_R_ (breath/min)	52.4 ± 5.6	56.8 ± 8.8	0.61	0.04
*V*_Epeak_ (L/min)	102.4 ± 13.5	118.7 ± 13.3	1.24	0.002
*V*_A_ (L/min)	91.1 ± 9.0	106.2 ± 11.7	1.19	0.003
*V*_E_/*V*o_2_	24.7 ± 2.7	27 ± 2.9	1.69	< 0.001
*V*_E_/*V*co_2_	22.9 ± 2.4	24.7 ± 2.6	1.86	< 0.001
P_ETco2_ (mmHg)	36.0 ± 3.1	33.5 ± 3.3	1.13	0.004
SV (ml)	137.5 ± 34.0	137.8 ± 32.1	0.05	0.99
HR (beats/min)	189.2 ± 7.9	190.1 ± 7.7	0.18	0.56
CO (L/min)	25.4 ± 7.0	24.8 ± 5.0	0.07	0.82
∫Pm (cmH_2_O/min)	166.3 ± 29.1	138.5 ± 15.3	0.95	0.02
SpO_2_ (%)	94.2 ± 2.4	93.5 ± 1.9	0.29	0.35
Time to exhaustion (s)	944.1 ± 37.6	970.7 ± 50.4	0.80	0.02

CFM, cloth face masked trial; CON, unmasked control trial; *V*o_2peak_, peak oxygen uptake; *V*co_2peak_, peak carbon dioxide elimination; VT, tidal volume; *f*_R_, respiratory frequency; *V*_Epeak_, peak minute ventilation; *V*_A_, alveolar ventilation: P_ETco2_, end-tidal partial pressure of CO_2_; SV, stroke volume; HR, heart rate; CO, cardiac output; ∫Pm, the integral absolute value of mouth pressure; SpO_2_, arterial oxy-hemoglobin saturation. Values are expressed as the mean ± SD.

**Figure 2:**
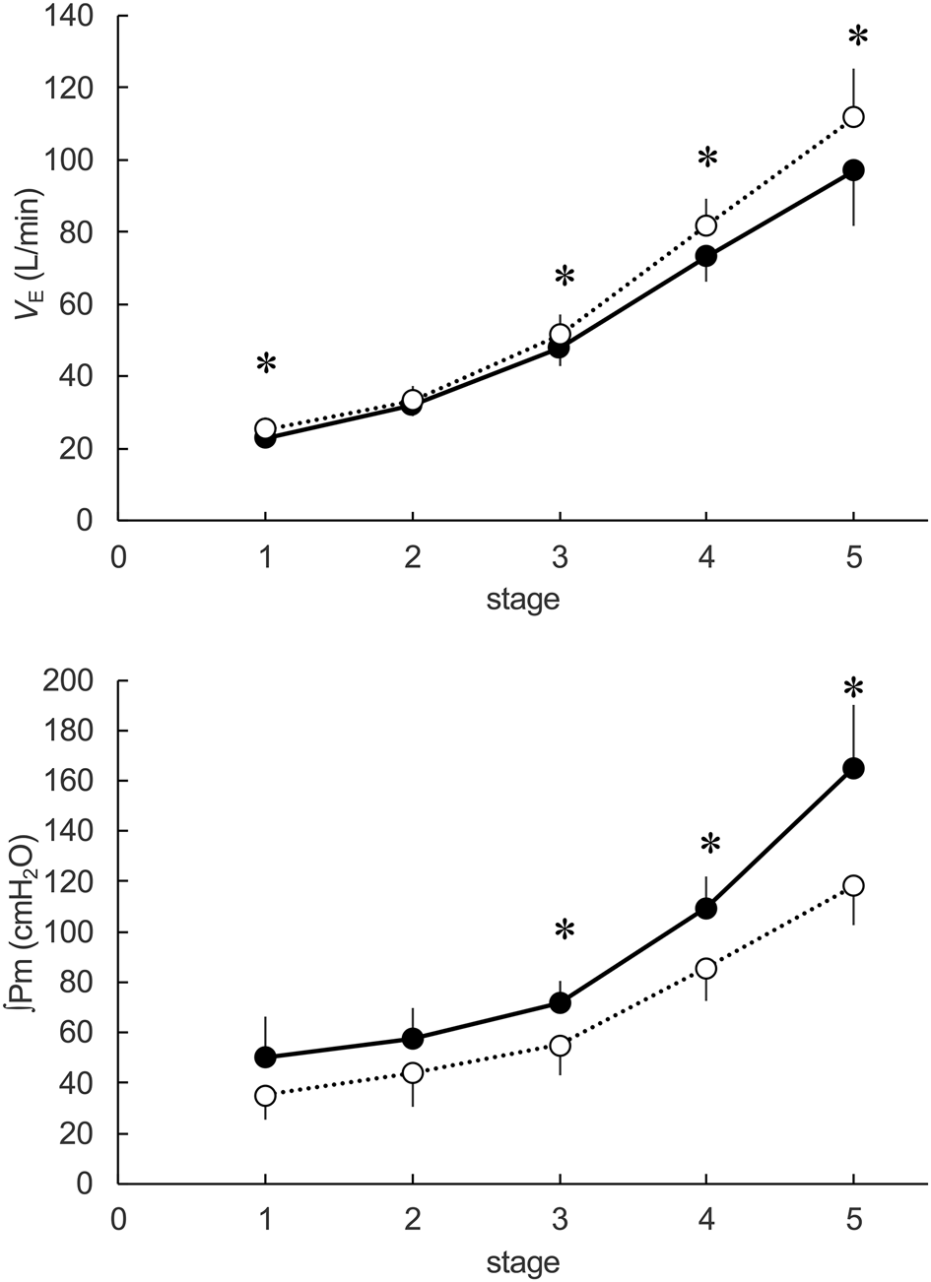
Minute ventilation and respiratory effort during incremental running test. The data show the minute ventilation (*V*_E_, upper panel) and minute integral of the mouth pressure (∫pm, lower panel). The black circles represent the cloth face masked (CFM) condition. The white circles indicate the unmasked control (CON) condition. There was a significant interaction (mask × stage) for both *V*_E_ (*P* = 0.01, pη^2^ = 0.47) and ∫pm (*P* = 0.01, pη^2^ = 0.51). *, *P* < 0.05, between the FM and CON conditions in the post hoc test.

Table 3 presents variables for each stage during the incremental treadmill test. There was no significant interaction for *V*o_2_ (*P* = 0.14, ηp^2^ = 0.20) and carbon dioxide emission (*V*co_2_) (*P* = 0.09, ηp^2^ = 0.25). However, there was a significant main effect for the mask factor on the *V*co_2_ (*P* = 0.04, ηp^2^ = 0.24). A significant interaction was observed in *V*_E_ (*P* = 0.01, ηp^2^ = 0.47) (Fig. 2). Regarding respiratory pattern, while the VT showed no significant main effect in the CFM condition (*P* = 0.32, ηp^2^ = 0.09), *f*_R_ showed a significant main effect in the CFM condition (*P* < 0.001, ηp^2^ = 0.67). *V*_A_ also showed a significant interaction (*P* = 0.01, ηp^2^ = 0.01). There was a significantly greater interaction in ∫Pm (*P* = 0.01, ηp^2^ = 0.51) in the CFM condition than in the CON condition, up to the third stage (Fig. 2). However, there was no significant interaction for both peak inspiratory mouth pressure (PI_peak_) and peak expiratory mouth pressure (PE_peak_) (*P* = 0.19, ηp^2^ = 0.20 and *P* = 0.05, ηp^2^ = 0.41). Regarding SpO_2_, there was a significant main effect for the stage factor, it gradually decreased with intensity-dependently (*P* < 0.001, ηp^2^ = 0.81); however, there was no significant effect for the mask factor. Further, there was no effect in cardiac responses for the mask factor.

**Table 3:** Variables for submaximal intensity.

		Stage					ANOVA		
		1	2	3	4	5	mask	stage	interaction
*V*o_2_ (mL/min)	CFM	1125 ± 85	1494 ± 133	2259 ± 120	3123 ± 185	3481 ± 385	P = 0.26	P < 0.001	P = 0.14
	CON	1130 ± 97	1487 ± 130	2271 ± 209	3231 ± 314	3706 ± 465	ηp^2^ = 0.12	ηp^2^ = 0.98	ηp^2^ = 0.20
*V*co_2_ (mL/min)	CFM	810 ± 9	1189 ± 124	1972 ± 144	3024 ± 182	3703 ± 348	P = 0.04	P < 0.001	P = 0.09
	CON	851 ± 54	1220 ± 97	2055 ± 156	3212 ± 269	3989 ± 503	ηp^2^ = 0.24	ηp^2^ = 0.98	ηp^2^ = 0.25
VT (L)	CFM	1.1 ± 0.2	1.4 ± 0.2	1.8 ± 0.1	2.0 ± 0.2	2.0 ± 0.3	P = 0.32	P < 0.001	P = 0.08
	CON	1.1 ± 0.2	1.3 ± 0.2	1.8 ± 0.3	2.1 ± 0.3	2.2 ± 0.3	ηp^2^ = 0.09	ηp^2^ = 0.90	ηp^2^ = 0.26
*f*_R_ (breath/min)	CFM	22.4 ± 3.7	23 ± 4.2	27.4 ± 3.1	36.9 ± 4.9	49.4 ± 6.6	P < 0.001	P < 0.001	P = 0.82
	CON	23.9 ± 4.5	27.1 ± 4.1	29.1 ± 4.5	38.9 ± 5	52.5 ± 10	ηp^2^ = 0.67	ηp^2^ = 0.92	ηp^2^ = 0.00
*V*_E_ (L/min)	CFM	23.0 ± 3.1*	31.8 ± 4.1	47.8 ± 5.5*	73.2 ± 7.4*	97.1 ± 13.7*	P = 0.00	P < 0.001	P = 0.01
	CON	25.3 ± 1.6	33.3 ± 2.7	51.5 ± 5	81.8 ± 6.9	111.8 ± 15.2	ηp^2^ = 0.60	ηp^2^ = 0.98	ηp^2^ = 0.47
P_ET_co_2_ (mmHg)	CFM	33 ± 2.7	31.2 ± 4.1	36.4 ± 4.5	39.5 ± 3.9	37 ± 3.1	P < 0.001	P < 0.001	P = 0.83
	CON	30.9 ± 2.6	29.7 ± 2.6	35.4 ± 5.2	37.6 ± 3.5	34.9 ± 3.9	ηp^2^ = 0.74	ηp^2^ = 0.74	ηp^2^ = 0.00
PI_peak_ (cmH_2_O)	CFM	− 2.75 ± 1.56	− 3.31 ± 1.93	− 3.70 ± 2.21	− 4.08 ± 2.54	− 4.30 ± 2.39	P = 0.19	P < 0.001	P = 0.05
	CON	− 1.28 ± 1.27	− 1.52 ± 1.52	− 1.78 ± 1.90	− 2.65 ± 1.31	− 4.63 ± 4.28	ηp^2^ = 0.20	ηp^2^ = 0.81	ηp^2^ = 0.39
PE_peak_ (cmH_2_O)	CFM	2.00 ± 1.98	2.37 ± 1.65	4.85 ± 2.43	9.37 ± 4.69	11.47 ± 5.51	P = 0.05	P = 0.03	P = 0.27
	CON	2.55 ± 1.37	3.04 ± 1.21	4.77 ± 1.85	7.13 ± 2.82	8.28 ± 3.83	ηp^2^ = 0.41	ηp^2^ = 0.45	ηp^2^ = 0.15
SV (mL)	CFM	100.3 ± 19.8	104.7 ± 18.4	109.0 ± 20.3	116.9 ± 23.9	134.1 ± 33.6	P = 0.81	P < 0.001	P = 0.43
	CON	96.2 ± 12.8	106.6 ± 15	113.8 ± 21.4	119 ± 22.6	120.8 ± 26.6	ηp^2^ = 0.00	ηp^2^ = 0.78	ηp^2^ = 0.06
HR (beat/min)	CFM	96.9 ± 12.0	114.6 ± 11.2	143.6 ± 12.0	173.6 ± 10.8	181.9 ± 22.5	P = 0.92	P < 0.001	P = 0.68
	CON	97.6 ± 8.6	112.9 ± 8.8	144.5 ± 11.2	169.2 ± 13.8	185.8 ± 10.4	ηp^2^ = 0.00	ηp^2^ = 0.98	ηp^2^ = 0.01
CO (L/min)	CFM	9.7 ± 2.0	11.9 ± 2.1	15.6 ± 2.6	20.3 ± 4.3	24.5 ± 7.4	P = 0.72	P < 0.001	P = 0.45
	CON	9.4 ± 1.1	12 ± 1.7	16.3 ± 2.5	19.9 ± 2.7	22.3 ± 4.3	ηp^2^ = 0.01	ηp^2^ = 0.94	ηp^2^ = 0.05
SpO_2_ (%)	CFM	98.9 ± 1.0	99.0 ± 1.0	98.5 ± 1.2	97.3 ± 1.3	94.2 ± 2.3	P = 0.37	P < 0.001	P = 0.07
	CON	99.5 ± 0.8	98.9 ± 1	98.5 ± 1.4	96.3 ± 2.5	93.9 ± 2	ηp^2^ = 0.08	ηp^2^ = 0.81	ηp^2^ = 0.30

CFM, cloth face masked trial; CON, unmasked control trial; *V*o_2_, oxygen uptake; *V*co_2_, carbon dioxide elimination; VT, tidal volume; *f*_R_, respiratory frequency; *V*_E_, minute ventilation; P_ETco2_, end-tidal partial pressure of CO_2_; PI_peak_, peak inspiratory mouth pressure; PE_peak_, peak expiratory mouth pressure; SV, stroke volume; HR, heart rate; CO, cardiac output; ∫Pm, the integral absolute value of mouth pressure; SaO_2_, arterial oxy-hemoglobin saturation. Values are expressed as the mean ± SD. *,* P *< 0.05, significantly different to the CON condition.

**Figure 3:**
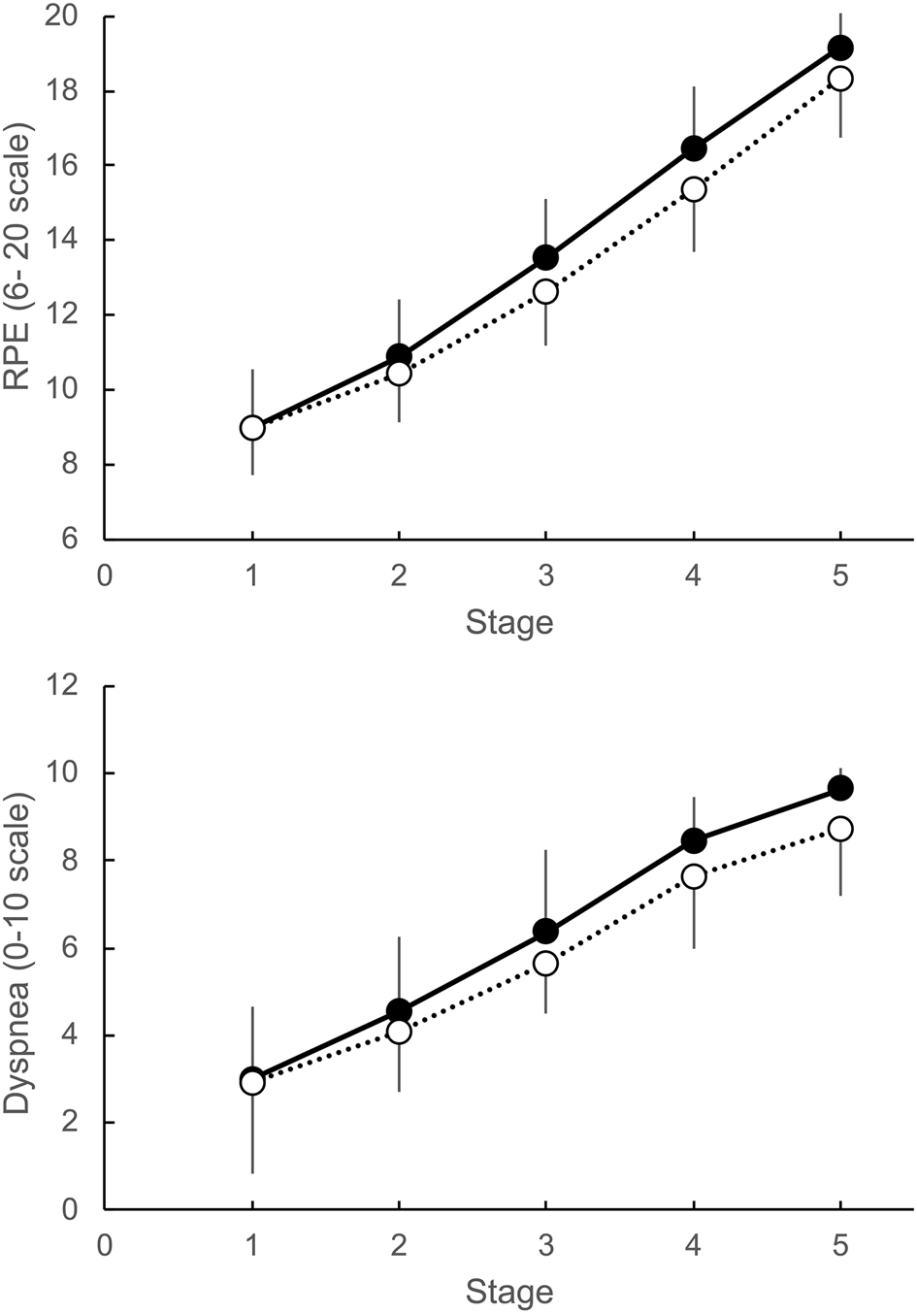
RPE and Dyspnea during incremental running test. Data show RPE [6–20 scale] (upper panel) and dyspnea [1–10 scale] (lower panel). The black circles represent the cloth face masked (CFM) condition. The white circles indicate the unmasked control (CON) condition. There were no significant main effects of the mask factor on either RPE (*P* = 0.09, ηp^2^ = 0.25) or dyspnea (*P* = 0.20, ηp^2^ = 0.15). RPE and dyspnea tended to be higher in the FM condition than in the Unmask condition, but the differences were not significant (*P* = 0.14, ηp^2^ = 0.19, *P* = 0.06, ηp^2^ = 0.30).

RPE and dyspnea were not significantly different between the two conditions (*P* = 0.14, ηp^2^ = 0.19 and *P* = 0.06, ηp^2^ = 0.30, respectively) (Fig. 3).

## Discussion

Greater airflow resistance while wearing a face mask could be considered an important factor influencing physiological responses during exercise. Cloth face masks have lower airflow resistance than surgical masks^14^. Thus, the focus of the present study was to examine cardiorespiratory responses and respiratory effort during exercise while wearing cloth face masks, such as those used in sports. Our novel findings were that the exercise pulmonary ventilation was reduced with a cloth face mask, while resistive respiratory muscle work was increased. Furthermore, the central circulatory system was unaffected. SpO_2_ and *V*O_2peak_ also did not decrease with the CFM condition compared with the CON condition. Further, the cloth face mask had a slightly significant negative effect on exercise tolerance. Therefore, we can conclude that a thinner cloth mask in healthy young men affects respiratory responses but does not induce a decrease in SpO_2_ or *V*o_2_, despite the slight decrease in exercise tolerance.

Previous studies have reported a significant decrease in *V*_E_ during incremental running tests using surgical masks^5,6,8,23^. Our results of *V*_E_ are consistent with those of previous studies. Among our participants, the lower *V*_E_ with a cloth face mask was more pronounced above the third stage. During high airflow at a high *V*_E_, turbulence in the airways and mouth is more prevalent, in turn increasing the flow resistance, which is a limiting factor for *V*_E_^27^. In this study, the PI_peak_ and PE_peak_ during exercises above moderate intensity were 2–3 cmH_2_O greater in the CFM condition than in the CON condition; however, it was not significant (*P* = 0.19 in PI_peak_, and *P* = 0.05 in PE_peak_). Furthermore, the ∫Pm was significantly higher from the third stage onwards. A higher P_ET_co_2_ in the CFM condition, even a slight elevation, should produce a hyperventilation demand by chemo-reflex^28^. Thus, we predict that wearing a cloth face mask suppresses *V*_E_ due to the increased airflow resistance, despite the high ventilatory demand by carbon dioxide rebreathing.

Previous studies have not observed a decrease in SpO_2_, even with the use of surgical masks^11,13,29^. Inadequate pulmonary hyperventilation impairs alveolar gas exchange and potentially contributes to decreased SaO_2_^15,30,31^. However, this is not the case for all participants. For instance, in untrained individuals, SaO_2_ is maintained during intensive exercise even without a face mask, and the amount of pulmonary ventilation does not affect gas exchange in normal lungs^30^. In contrast, among individuals who experience exercise-induced arterial hypoxemia (EIAH), the height of* V*_E_ can affect SaO_2_^15^. In addition, such individuals have a greater decrease in SpO_2_ and *V*o_2max_ with hypoxic gas breathing^32^. These indicate that the importance of hyperventilatory response to the partial pressure of oxygen is more remarkable in individuals with EIAH^15,31,32^. Our participants showed an SpO_2_ of 93% at maximal exercise, which was lower than that at the first stage (99%). This 6% decrease in SpO_2_ was assumed to have provoked a mild EIAH. Thus, we could have expected that the effect of wearing a face mask would be considerable among individuals with EIAH. *V*_E_ was reduced by 20%, and *V*_A_ was also significantly reduced. However, wearing a cloth face mask did not induce any additional reduction in SpO_2_. The decrease in *V*_E_ while wearing a cloth face mask was associated with a decrease in *f*_R_, while VT was maintained. This study did not measure the lung volume during exercise; however, it was speculated that maintaining VT with a cloth face mask would not alter the dead space to VT ratio and end-expiratory lung volume, and thus would impact the gas exchange less^33^.

We aimed to reveal the effects of wearing a cloth face mask on the central circulatory response. Whole-body blood flow, and in turn, CO did not change even though respiratory resistance increased and workload in the respiratory muscles increased by approximately 1.5 times^17^. In this study, although the estimated respiratory muscle work increased by approximately 20%, CO did not differ between the CFM and CON conditions, consistent with a previous study that compared surgical mask and N95 respirator^7^. A surgical mask increases the HR during submaximal exercise^5^, whereas no difference in HR has been reported during high-intensity exercise. However, in our study, there was no difference in HR, SV, and CO during the incremental exercise under both conditions. It was suggested that the central circulation burden was not higher when wearing the mask, and that the relative physiological intensity was not affected.

Deriver et al.^20^ reported a decrease in *V*o_2peak_ while exercising with a cloth face mask; Umutlu et al.^6^ also reported a decrease in *V*o_2peak_ while exercising with a surgical mask. The decrease in *V*o_2peak_ in the previous study was not associated with a decrease in SpO_2_. Furthermore, in our participants, the SpO_2_ was maintained, and CO was unaffected at maximal running even when wearing the cloth face mask. This implies that the oxygen supply was maintained, and consequently, *V*o_2peak_ was not significantly different with and without a cloth face mask. However, the statistical effect size was large, and the extent of *V*o_2peak_ decrease in the CFM condition was 4.4%. The characteristics of the participants may explain the inconsistency between our results and those of previous studies^6,20^. The participants in previous studies included sedentary patients, women, and older patients, and their *V*o_2max_ was lower than those of our participants. Additionally, sex and age affect the impact of wearing face masks^23^. Our results implied that apparently healthy young men with higher physical fitness might be less susceptible to the negative effects of wearing cloth face masks.

Consistent with previous studies^6,20,23^, our results demonstrated that wearing a mask slightly impaired exercise tolerance, suggesting that exercise performance was affected even with low-resistance cloth face masks. We did not observe a significant decrease in *V*o_2peak_ in the CFM condition. However, the *V*o_2peak_ decrease in the CFM condition was 4.4%. Thus, the decrease in *V*o_2peak_ may be due to decreased exercise tolerance. Driver et al.^20^ reported that exercise tolerance was impaired with a substantial decrease in *V*o_2peak_ and peak HR. Therefore, under the exercise protocol employed by Driver et al.^20^ and our study, the *V*o_2peak_ and peak HR may result from a decrease in exercise tolerance due to other factors and not a factor that decreases exercise tolerance^34^.

The potential mechanisms underlying decreased exercise tolerance can be attributed to the effect of the respiratory flow resistance due to the mask filter. Furthermore, increasing dead space or wearing a resistive training mask during exercise increases respiratory effort, leading to increased dyspnea and decreased exercise tolerance^35,36^. However, in the present study, dyspnea tended to be higher in the CFM condition, but the difference was not statistically significant. We evaluated resisted respiratory effort during exercise using mouth cavity pressure; higher respiratory muscle activity with a cloth face mask was not observed in the earlier stages but became significant in the later stages. This may be related to the increased turbulence in the airway being facilitated by the increased airflow. It can be assumed that our participants had a high ventilatory effort in the CFM condition, even though the flow resistance was lower than that of a surgical mask and N95 respirator^14^. During maximal exercise, *V*o_2_ for respiratory muscle activity accounts for a significant proportion of whole-body *V*o_2_ even without wearing a face mask^37^, causing blood flow competition between the respiratory muscles and active muscles^16,17^. Increased respiratory muscle activity or fatigue during exercise causes respiratory muscle-induced metaboreflex, leading to limb vasoconstriction^38^ and blood flow restriction^39^. This is postulated to be a factor that limits exercise performance owing to respiratory muscle work^18^.

There were some limitations in this study. First, the participants were healthy young men. It would be hasty to conclude from our results that a cloth face mask reduces exercise performance but does not affect the oxygen uptake in all populations. Different outcomes will likely be obtained for different patient populations and those with various respiratory conditions, such as children, older patients, and patients with chronic obstructive pulmonary disease.

Second, in this study, the participants performed an exercise test with a gas collection mask for gas analysis connected to the face mask. Therefore, although the gas sampling mask provides a lower flow resistance, it still imposes a higher respiratory load than that during a regular workout. Hence, the results obtained in this study could have overestimated the effect of cloth face masks.

Third, to ensure the reliability of the impedance cardiograph variables, we presented values averaged over 60 s and did the same for the gas analysis variables. Therefore, the data may be less sensitive than those obtained over a shorter period^40^. However, no significant differences were confirmed between 30 and 60 s averaged data.

Finally, the exercise protocol employed included a simultaneous increase in speed and incline, which could have resulted in premature muscle fatigue and terminated the exercise before *V*o_2_ reached a maximum; thus, the *V*o_2peak_ in this study may not have assessed aerobic capacity. Further, we did not perform a verification phase test^41^; therefore, we could not detect *V*o_2max_. Despite these limitations, we can conclude that in our incremental treadmill running test, impairing hyperventilation with CFM impacts *V*o_2_ less.

## Data Availability

Due to ethical committee recommendations, supporting data is not fully available. However, some calculated data supporting this study's findings are available from the corresponding author upon reasonable request.
